# Supplementary material for: Individual characteristics outperform resting-state fMRI for the prediction of behavioral phenotypes
Source: Commun Biol. 2024 Jun 26;7:771. doi: 10.1038/s42003-024-06438-5 (PMC11208538; doi:10.1038/s42003-024-06438-5)
Supplement: Supplementary file 2 — Supplementary Information [file 42003_2024_6438_MOESM2_ESM.pdf]

Supplementary Materials

Table S1: RsfMRI features and the prediction targets in this study.

RsfMRI Feature name	Category	Spatial resolution
Weighted permutation entropy (wPE)	Temporal complexity	ROI-wise
Range entropy ($RangeEn_B$)	Temporal complexity	ROI-wise
Multiscale entropy (MSE)	Temporal complexity	ROI-wise
Hurst exponent (HE)	Temporal complexity	ROI-wise
Eigenvector centrality (EC)	Functional connectivity	ROI-wise
Weighted clustering coefficient (wCC)	Functional connectivity	ROI-wise
Fractional amplitude of low-frequency fluctuations (fALFF)	Functional connectivity	Voxel-wise
Local correlation (LCOR)	Functional connectivity	Voxel-wise
Global correlation (GCOR)	Functional connectivity	Voxel-wise
**Prediction target**	**UK Biobank Data field**	
Fluid intelligence	20016	
Processing time	20023	
Visual memory	399	
Numeric memory	20240	
Fish consumer yesterday	103140	

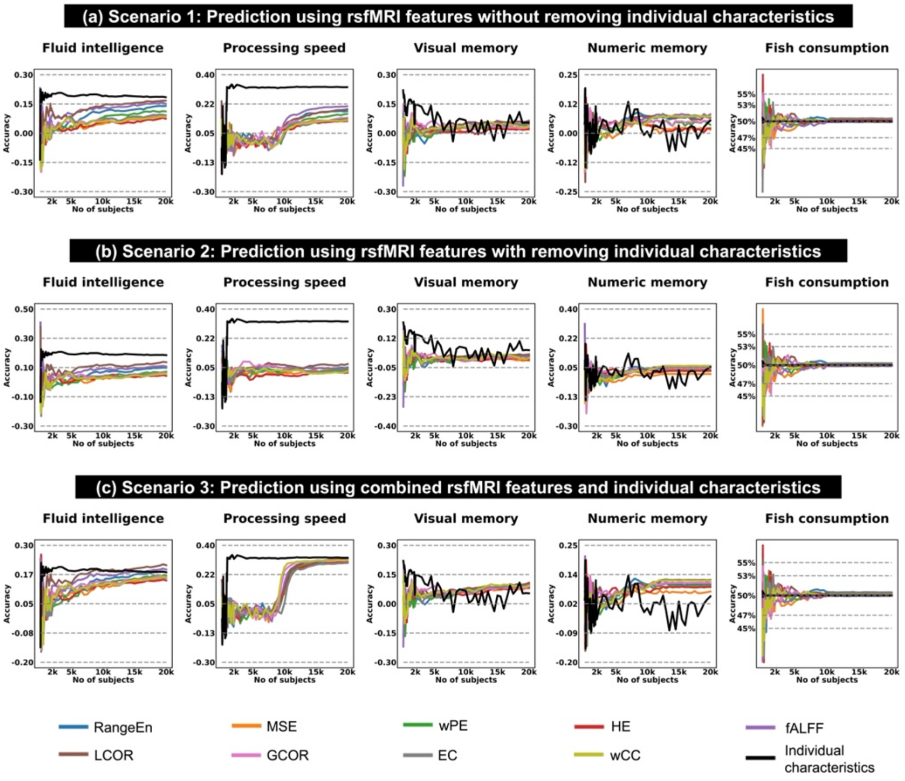

Figure S1: Prediction accuracy scores associated with nine rsfMRI features and five prediction targets using scenarios 1–3 of this study using the Schaefer400 brain atlas and linear SVM predictive modeling (see also figures 1-b.1–b.3 and Methods). The prediction accuracies of individual characteristics only (Scenario 4 in figure 1-b.4) have been plotted in bold black on all panels. Prediction accuracies of the fluid intelligence, processing speed, visual memory, and numeric memory scores are computed as the Spearman correlation between the actual values and predicted values through SVM modeling. The prediction accuracy of Fish consumer yesterday is computed as the balanced accuracy through SVM binary classification. Each rsfMRI feature is illustrated in a distinct color and listed in the figure legend. In each figure panel, the x-axis represents the population size in the analysis, and the y-axis shows the prediction accuracy. The predictive modeling of each pair of features and targets is repeated for different sample sizes in the UK Biobank, ranging from $N_{subject}$ = 100 to $N_{subject}$ = 20,000. The population sizes from 100 to 2000 were increased with a 50-step increment and from 2000 to 20,000 with a 500-step increment. See figure S8 for the boxplot representation of these results. Abbreviations: rsfMRI = resting state functional magnetic resonance imaging, RangeEn = range entropy, MSE = multiscale entropy, wPE = weighted permutation entropy, HE = Hurst exponent, fALFF = fractional amplitude of low frequency fluctuations, LCOR = local correlation, GCOR = global correlation, EC = eigenvector centrality, wCC = weighted clustering coefficient, SVM = support vector machine.

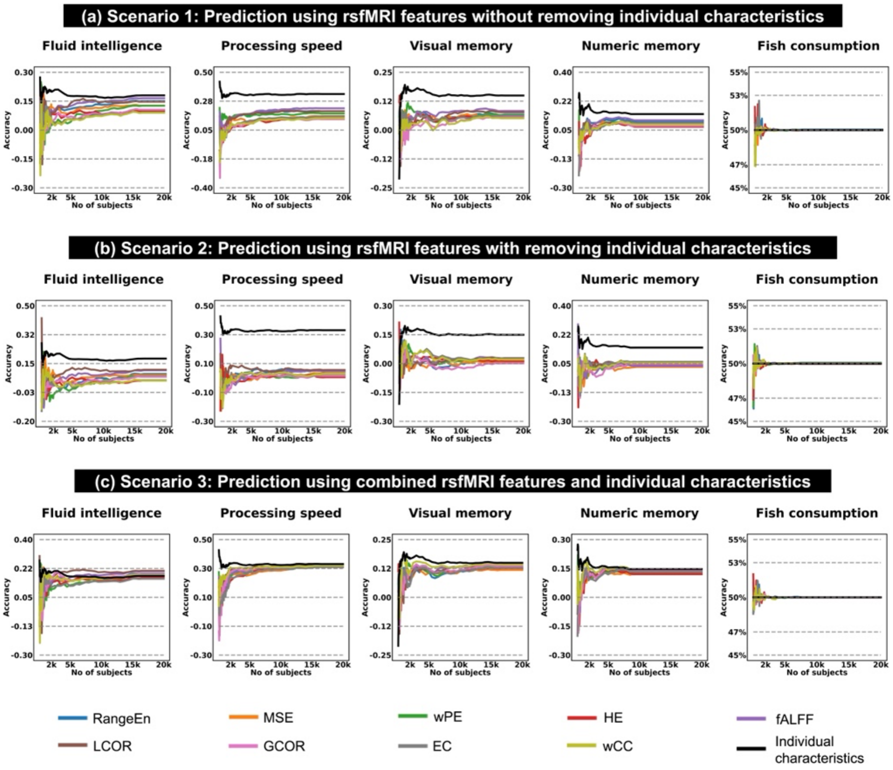

Figure S2: Prediction accuracy scores associated with nine rsfMRI features and five prediction targets using scenarios 1–3 of this study using the Glasser360 brain atlas and ridge predictive modeling (see also figures 1-b.1–b.3 and Methods). The prediction accuracies of individual characteristics only (Scenario 4 in figure 1-b.4) have been plotted in bold black on all panels. Prediction accuracies of the fluid intelligence, processing speed, visual memory, and numeric memory scores are computed as the Spearman correlation between the actual values and predicted values through ridge regression modeling. The prediction accuracy of Fish consumer yesterday is computed as the balanced accuracy through ridge binary classification. Each rsfMRI feature is illustrated in a distinct color and listed in the figure legend. In each figure panel, the x-axis represents the population size in the analysis, and the y-axis shows the prediction accuracy. The predictive modeling of each pair of features and targets is repeated for different sample sizes in the UK Biobank, ranging from $N_{subject}$ = 100 to $N_{subject}$ = 20,000. The population sizes from 100 to 2000 were increased with a 50-step increment and from 2000 to 20,000 with a 500-step increment. See figure S9 for the boxplot representation of these results. Abbreviations: rsfMRI = resting state functional magnetic resonance imaging, RangeEn = range entropy, MSE = multiscale entropy, wPE = weighted permutation entropy, HE = Hurst exponent, fALFF = fractional amplitude of low frequency fluctuations, LCOR = local correlation, GCOR = global correlation, EC = eigenvector centrality, wCC = weighted clustering coefficient.

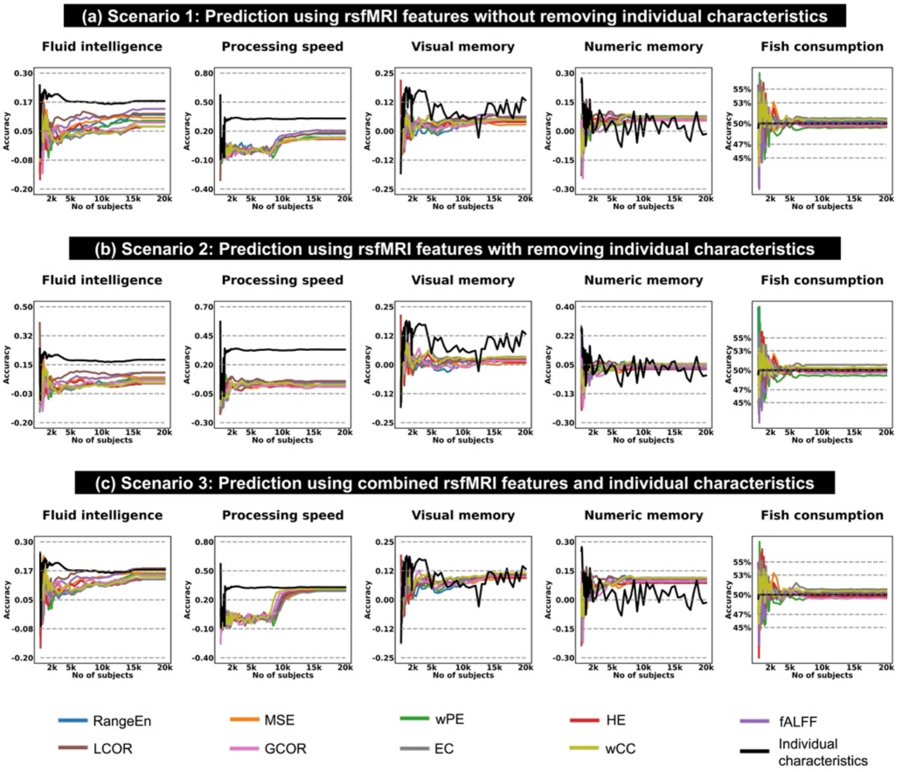

Figure S3: Prediction accuracy scores associated with nine rsfMRI features and five prediction targets using scenarios 1–3 of this study using the Glasser360 brain atlas and linear SVM predictive modeling (see also figures 1-b.1–b.3 and Methods). The prediction accuracies of individual characteristics only (Scenario 4 in figure 1-b.4) have been plotted in bold black on all panels. Prediction accuracies of the fluid intelligence, processing speed, visual memory, and numeric memory scores are computed as the Spearman correlation between the actual values and predicted values through SVM modeling. The prediction accuracy of Fish consumer yesterday is computed as the balanced accuracy through SVM binary classification. Each rsfMRI feature is illustrated in a distinct color and listed in the figure legend. In each figure panel, the x-axis represents the population size in the analysis, and the y-axis shows the prediction accuracy. The predictive modeling of each pair of features and targets is repeated for different sample sizes in the UK Biobank, ranging from $N_{subject}$ = 100 to $N_{subject}$ = 20,000. The population sizes from 100 to 2000 were increased with a 50-step increment and from 2000 to 20,000 with a 500-step increment. See figure S10 for the boxplot representation of these results. Abbreviations: rsfMRI = resting state functional magnetic resonance imaging, RangeEn = range entropy, MSE = multiscale entropy, wPE = weighted permutation entropy, HE = Hurst exponent, fALFF = fractional amplitude of low frequency fluctuations, LCOR = local correlation, GCOR = global correlation, EC = eigenvector centrality, wCC = weighted clustering coefficient, SVM = support vector machine.

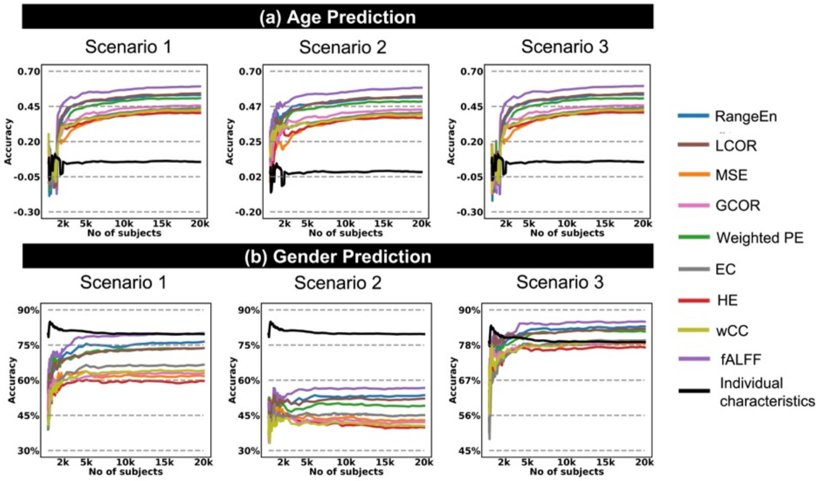

Figure S4: Prediction accuracy scores associated with nine rsfMRI features and age and gender as targets using scenarios 1–3 of this study using the Schaefer400 brain atlas and linear SVM predictive modeling (see also figures 1-b.1–b.3 and Methods). For age prediction, we considered gender and TIV as confounds, while for gender prediction, we considered age and TIV as confounds. Age prediction accuracies are computed as the Pearson correlation between the actual values and predicted values through SVM modeling. Gender prediction accuracies are computed as the balanced accuracy through SVM binary classification. Each rsfMRI feature is illustrated in a distinct color and listed in the figure legend. The population sizes from 100 to 2000 were increased with a 50-step increment and from 2000 to 20,000 with a 500-step increment. See figure S12 for the boxplot representation of these results. Abbreviations: rsfMRI = resting state functional magnetic resonance imaging, RangeEn = range entropy, MSE = multiscale entropy, wPE = weighted permutation entropy, HE = Hurst exponent, fALFF = fractional amplitude of low frequency fluctuations, LCOR = local correlation, GCOR = global correlation, EC = eigenvector centrality, wCC = weighted clustering coefficient, SVM = support vector machine.

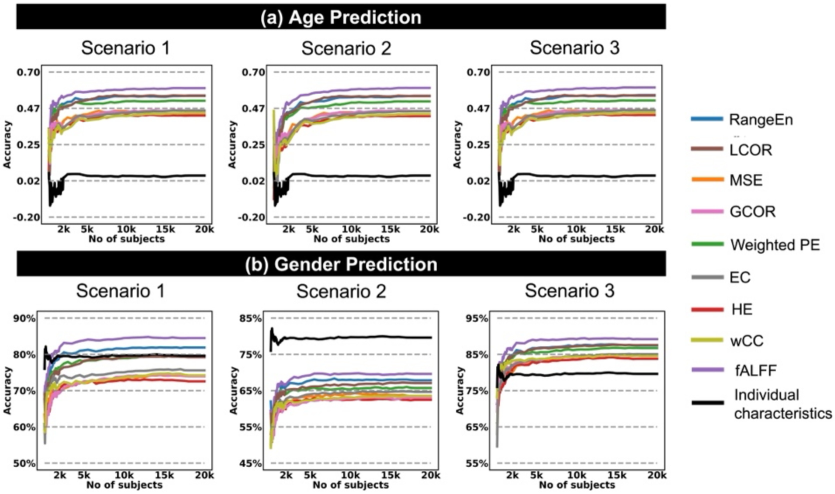

Figure S5: Prediction accuracy scores associated with nine rsfMRI features and age and gender as targets using scenarios 1–3 of this study using the Glasser360 brain atlas and ridge predictive modeling (see also figures 1-b.1–b.3 and Methods). For age prediction, we considered gender and TIV as confounds, while for gender prediction, we considered age and TIV as confounds. Age prediction accuracies are computed as the Pearson correlation between the actual values and predicted values through ridge regression modeling. Gender prediction accuracies are computed as the balanced accuracy through ridge binary classification. Each rsfMRI feature is illustrated in a distinct color and listed in the figure legend. The population sizes from 100 to 2000 were increased with a 50-step increment and from 2000 to 20,000 with a 500-step increment. See figure S13 for the boxplot representation of these results. Abbreviations: rsfMRI = resting state functional magnetic resonance imaging, RangeEn = range entropy, MSE = multiscale entropy, wPE = weighted permutation entropy, HE = Hurst exponent, fALFF = fractional amplitude of low frequency fluctuations, LCOR = local correlation, GCOR = global correlation, EC = eigenvector centrality, wCC = weighted clustering coefficient.

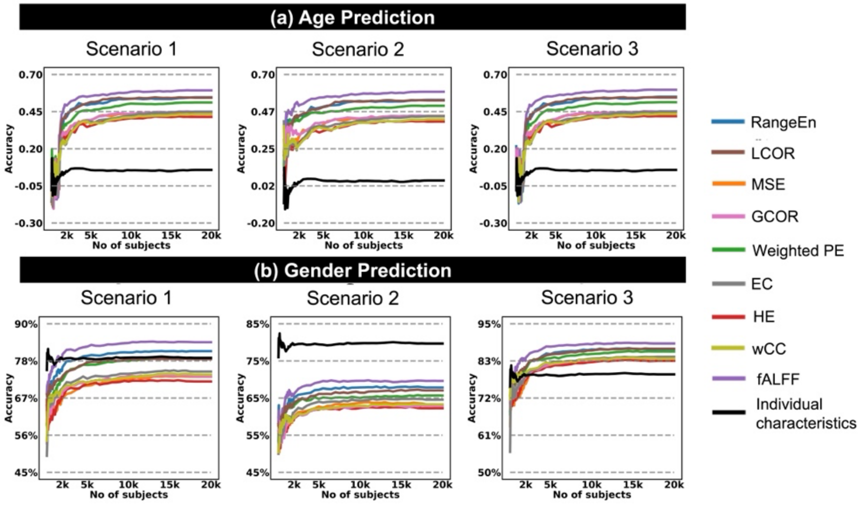

Figure S6: Prediction accuracy scores associated with nine rsfMRI features and age and gender as targets using scenarios 1–3 of this study using the Glasser360 brain atlas and linear SVM predictive modeling (see also figures 1-b.1–b.3 and Methods). For age prediction, we considered gender and TIV as confounds, while for gender prediction, we considered age and TIV as confounds. Age prediction accuracies are computed as the Pearson correlation between the actual values and predicted values through SVM modeling. Gender prediction accuracies are computed as the balanced accuracy through SVM binary classification. Each rsfMRI feature is illustrated in a distinct color and listed in the figure legend. The population sizes from 100 to 2000 were increased with a 50-step increment and from 2000 to 20,000 with a 500-step increment. See figure S14 for the boxplot representation of these results. Abbreviations: rsfMRI = resting state functional magnetic resonance imaging, RangeEn = range entropy, MSE = multiscale entropy, wPE = weighted permutation entropy, HE = Hurst exponent, fALFF = fractional amplitude of low frequency fluctuations, LCOR = local correlation, GCOR = global correlation, EC = eigenvector centrality, wCC = weighted clustering coefficient, SVM = support vector machine.

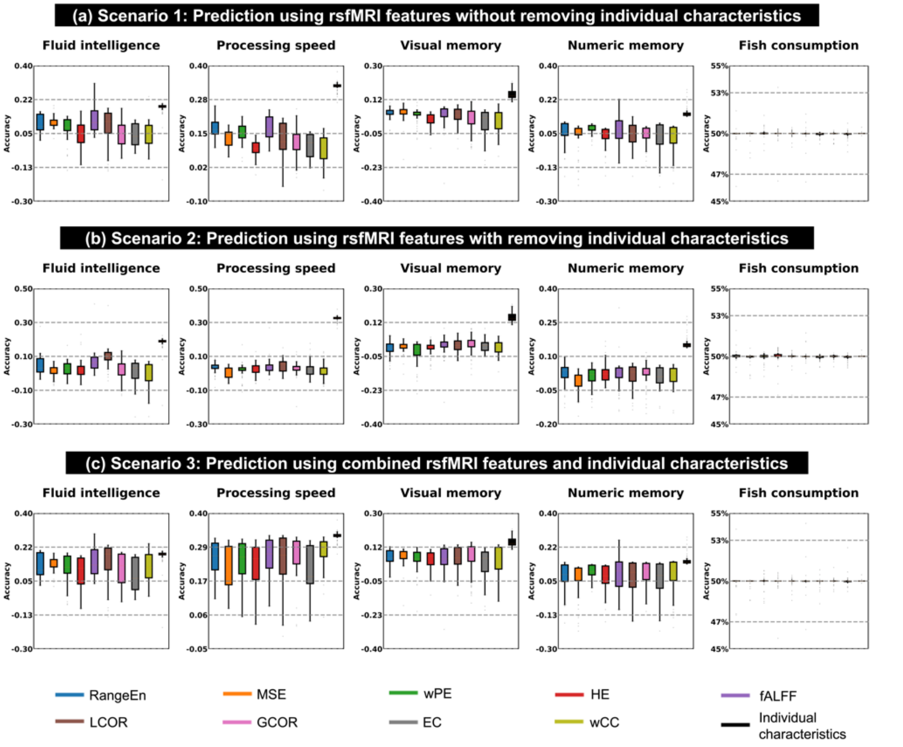

Figure S7: Prediction accuracy scores associated with nine rsfMRI features and five prediction targets using scenarios 1–3 of this study using the Schaefer400 brain atlas and ridge predictive modeling (see also figures 1-b.1–b.3 and Methods). Prediction accuracies of the fluid intelligence, processing speed, visual memory, and numeric memory scores are computed as the Pearson correlation between the actual values and predicted values through ridge regression modeling. The prediction accuracy of Fish consumer yesterday is computed as the balanced accuracy through ridge binary classification. Each rsfMRI feature is illustrated in a distinct color and listed in the figure legend. In each figure panel, the box has a line at the median and spans the complete range of sample sizes (from 100 to 20,000 participants), extending from the lower to upper quartile values of the prediction accuracies. The whiskers extend outside the box to display the data's range. The population sizes from 100 to 2000 were increased with a 50-step increment and from 2000 to 20,000 with a 500-step increment. See figure 2 for the representation of prediction accuracies over the range of sample sizes. Abbreviations: rsfMRI = resting state functional magnetic resonance imaging, RangeEn = range entropy, MSE = multiscale entropy, wPE = weighted permutation entropy, HE = Hurst exponent, fALFF = fractional amplitude of low frequency fluctuations, LCOR = local correlation, GCOR = global correlation, EC = eigenvector centrality, wCC = weighted clustering coefficient.

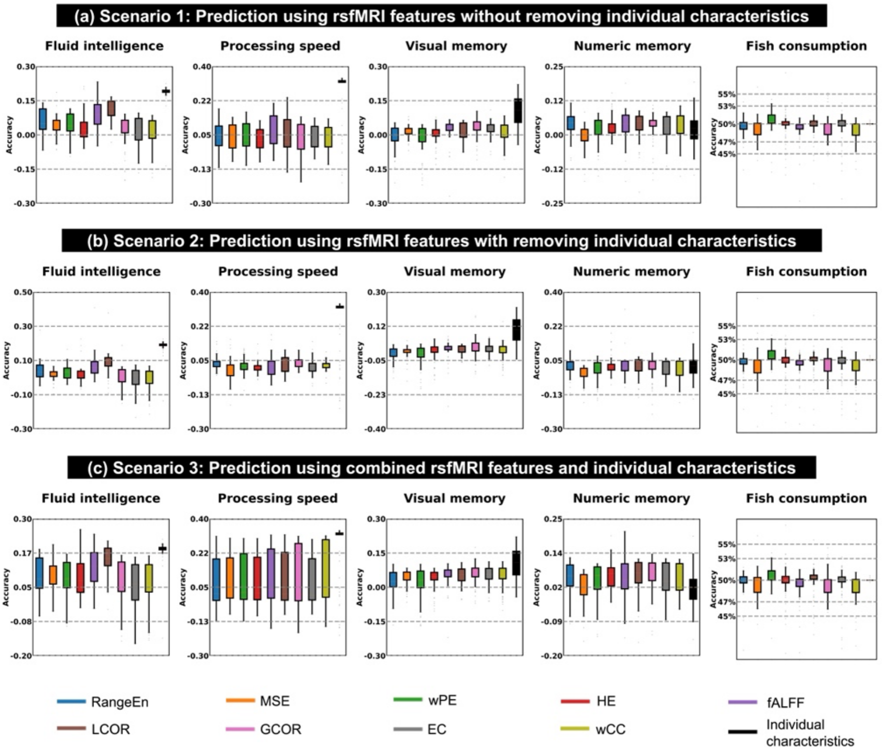

Figure S8: Prediction accuracy scores associated with nine rsfMRI features and five prediction targets using scenarios 1–3 of this study using the Schaefer400 brain atlas and linear SVM predictive modeling (see also figures 1-b.1–b.3 and Methods). Prediction accuracies of the fluid intelligence, processing speed, visual memory, and numeric memory scores are computed as the Pearson correlation between the actual values and predicted values through SVM modeling. The prediction accuracy of Fish consumer yesterday is computed as the balanced accuracy through SVM binary classification. Each rsfMRI feature is illustrated in a distinct color and listed in the figure legend. In each figure panel, the box has a line at the median and spans the complete range of sample sizes (from 100 to 20,000 participants), extending from the lower to upper quartile values of the prediction accuracies. The whiskers extend outside the box to display the data's range. The population sizes from 100 to 2000 were increased with a 50-step increment and from 2000 to 20,000 with a 500-step increment. See figure S1 for the representation of prediction accuracies over the range of sample sizes. Abbreviations: rsfMRI = resting state functional magnetic resonance imaging, RangeEn = range entropy, MSE = multiscale entropy, wPE = weighted permutation entropy, HE = Hurst exponent, fALFF = fractional amplitude of low frequency fluctuations, LCOR = local correlation, GCOR = global correlation, EC = eigenvector centrality, wCC = weighted clustering coefficient, SVM = support vector machine.

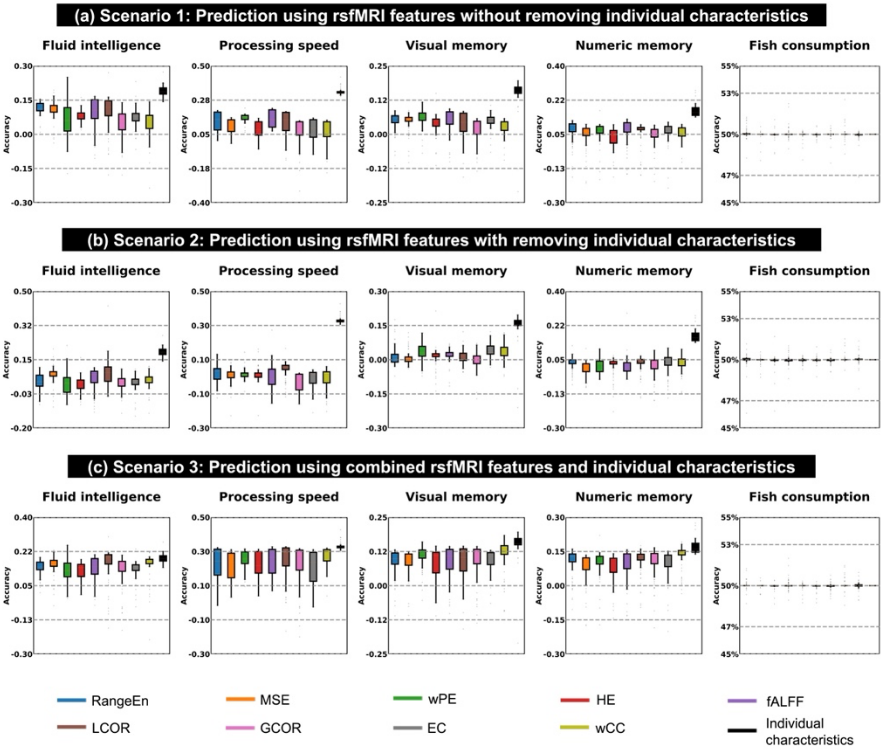

Figure S9: Prediction accuracy scores associated with nine rsfMRI features and five prediction targets using scenarios 1–3 of this study using the Glasser360 brain atlas and ridge predictive modeling (see also figures 1-b.1–b.3 and Methods). Prediction accuracies of the fluid intelligence, processing speed, visual memory, and numeric memory scores are computed as the Pearson correlation between the actual values and predicted values through ridge regression modeling. The prediction accuracy of Fish consumer yesterday is computed as the balanced accuracy through ridge binary classification. Each rsfMRI feature is illustrated in a distinct color and listed in the figure legend. In each figure panel, the box has a line at the median and spans the complete range of sample sizes (from 100 to 20,000 participants), extending from the lower to upper quartile values of the prediction accuracies. The whiskers extend outside the box to display the data's range. The population sizes from 100 to 2000 were increased with a 50-step increment and from 2000 to 20,000 with a 500-step increment. See figure S2 for the representation of prediction accuracies over the range of sample sizes. Abbreviations: rsfMRI = resting state functional magnetic resonance imaging, RangeEn = range entropy, MSE = multiscale entropy, wPE = weighted permutation entropy, HE = Hurst exponent, fALFF = fractional amplitude of low frequency fluctuations, LCOR = local correlation, GCOR = global correlation, EC = eigenvector centrality, wCC = weighted clustering coefficient.

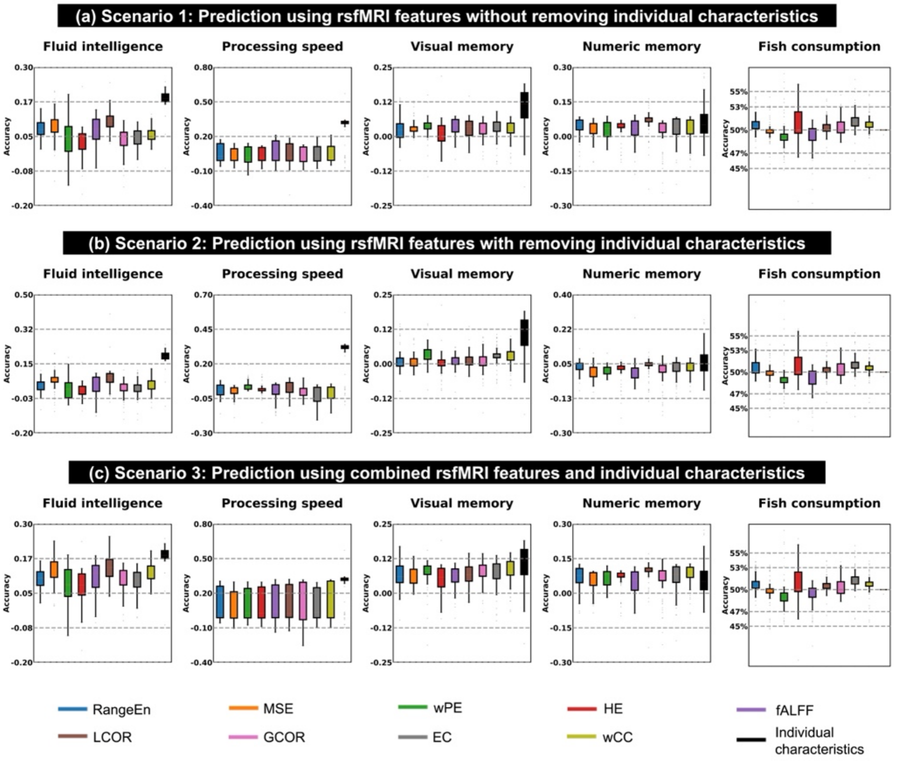

Figure S10: Prediction accuracy scores associated with nine rsfMRI features and five prediction targets using scenarios 1–3 of this study using the Glasser360 brain atlas and linear SVM predictive modeling (see also figures 1-b.1–b.3 and Methods). Prediction accuracies of the fluid intelligence, processing speed, visual memory, and numeric memory scores are computed as the Pearson correlation between the actual values and predicted values through SVM modeling. The prediction accuracy of Fish consumer yesterday is computed as the balanced accuracy through SVM binary classification. Each rsfMRI feature is illustrated in a distinct color and listed in the figure legend. In each figure panel, the box has a line at the median and spans the complete range of sample sizes (from 100 to 20,000 participants), extending from the lower to upper quartile values of the prediction accuracies. The whiskers extend outside the box to display the data's range. The population sizes from 100 to 2000 were increased with a 50-step increment and from 2000 to 20,000 with a 500-step increment. See figure S3 for the representation of prediction accuracies over the range of sample sizes. Abbreviations: rsfMRI = resting state functional magnetic resonance imaging, RangeEn = range entropy, MSE = multiscale entropy, wPE = weighted permutation entropy, HE = Hurst exponent, fALFF = fractional amplitude of low frequency fluctuations, LCOR = local correlation, GCOR = global correlation, EC = eigenvector centrality, wCC = weighted clustering coefficient, SVM = support vector machine.

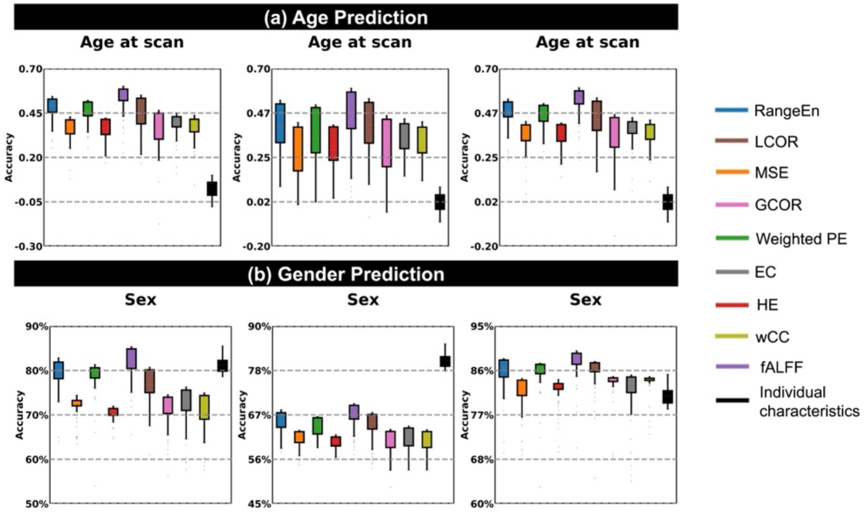

Figure S11: Prediction accuracy scores associated with nine rsfMRI features and age and gender as targets using scenarios 1–3 of this study using the Schaefer400 brain atlas and ridge predictive modeling (see also figures 1-b.1–b.3 and Methods). For age prediction, we considered gender and TIV as confounds, while for gender prediction, we considered age and TIV as confounds. Age prediction accuracies are computed as the Pearson correlation between the actual values and predicted values through ridge regression modeling. Gender prediction accuracies are computed as the balanced accuracy through ridge binary classification. Each rsfMRI feature is illustrated in a distinct color and listed in the figure legend. In each figure panel, the box has a line at the median and spans the complete range of sample sizes (from 100 to 20,000 participants), extending from the lower to upper quartile values of the prediction accuracies. The whiskers extend outside the box to display the data's range. The population sizes from 100 to 2000 were increased with a 50-step increment and from 2000 to 20,000 with a 500-step increment. See figure 4 for the representation of prediction accuracies over the range of sample sizes. Abbreviations: rsfMRI = resting state functional magnetic resonance imaging, TIV = total intracranial volume, RangeEn = range entropy, MSE = multiscale entropy, wPE = weighted permutation entropy, HE = Hurst exponent, fALFF = fractional amplitude of low frequency fluctuations, LCOR = local correlation, GCOR = global correlation, EC = eigenvector centrality, wCC = weighted clustering coefficient.

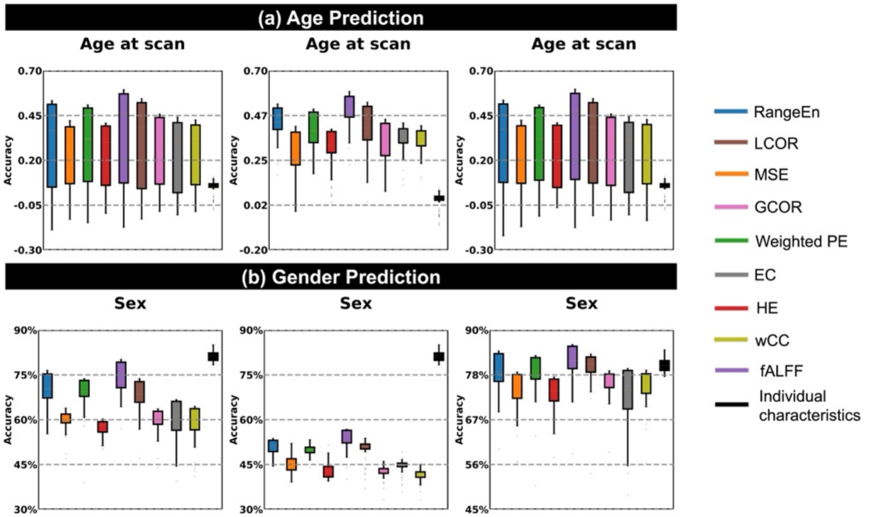

Figure S12: Prediction accuracy scores associated with nine rsfMRI features and age and gender as targets using scenarios 1–3 of this study using the Schaefer400 brain atlas and linear SVM predictive modeling (see also figures 1-b.1–b.3 and Methods). For age prediction, we considered gender and TIV as confounds, while for gender prediction, we considered age and TIV as confounds. Age prediction accuracies are computed as the Pearson correlation between the actual values and predicted values through SVM modeling. Gender prediction accuracies are computed as the balanced accuracy through SVM binary classification. Each rsfMRI feature is illustrated in a distinct color and listed in the figure legend. In each figure panel, the box has a line at the median and spans the complete range of sample sizes (from 100 to 20,000 participants), extending from the lower to upper quartile values of the prediction accuracies. The whiskers extend outside the box to display the data's range. The population sizes from 100 to 2000 were increased with a 50-step increment and from 2000 to 20,000 with a 500-step increment. See figure S4 for the representation of prediction accuracies over the range of sample sizes. Abbreviations: rsfMRI = resting state functional magnetic resonance imaging, TIV = total intracranial volume, RangeEn = range entropy, MSE = multiscale entropy, wPE = weighted permutation entropy, HE = Hurst exponent, fALFF = fractional amplitude of low frequency fluctuations, LCOR = local correlation, GCOR = global correlation, EC = eigenvector centrality, wCC = weighted clustering coefficient, SVM = support vector machine.

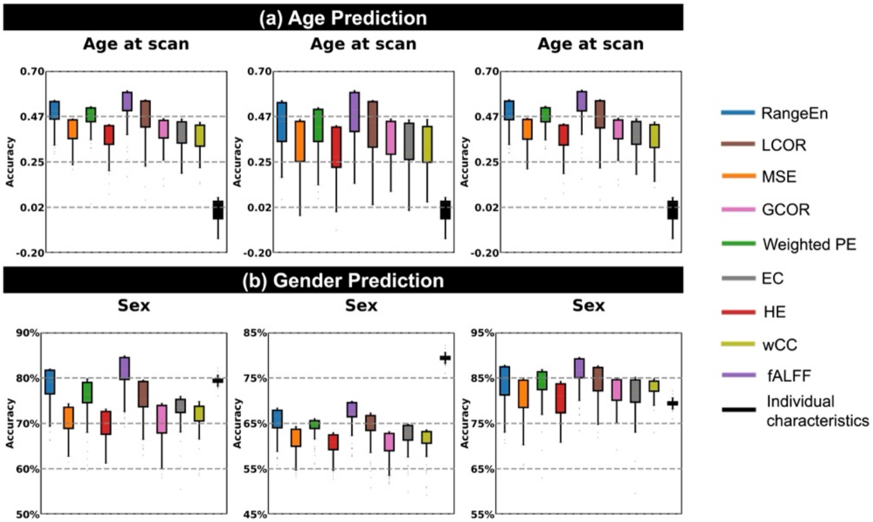

Figure S13: Prediction accuracy scores associated with nine rsfMRI features and age and gender as targets using scenarios 1–3 of this study using the Glasser360 brain atlas and ridge predictive modeling (see also figures 1-b.1–b.3 and Methods). For age prediction, we considered gender and TIV as confounds, while for gender prediction, we considered age and TIV as confounds. Age prediction accuracies are computed as the Pearson correlation between the actual values and predicted values through ridge regression modeling. Gender prediction accuracies are computed as the balanced accuracy through ridge binary classification. Each rsfMRI feature is illustrated in a distinct color and listed in the figure legend. In each figure panel, the box has a line at the median and spans the complete range of sample sizes (from 100 to 20,000 participants), extending from the lower to upper quartile values of the prediction accuracies. The whiskers extend outside the box to display the data's range. The population sizes from 100 to 2000 were increased with a 50-step increment and from 2000 to 20,000 with a 500-step increment. See figure S5 for the representation of prediction accuracies over the range of sample sizes. Abbreviations: rsfMRI = resting state functional magnetic resonance imaging, TIV = total intracranial volume, RangeEn = range entropy, MSE = multiscale entropy, wPE = weighted permutation entropy, HE = Hurst exponent, fALFF = fractional amplitude of low frequency fluctuations, LCOR = local correlation, GCOR = global correlation, EC = eigenvector centrality, wCC = weighted clustering coefficient.

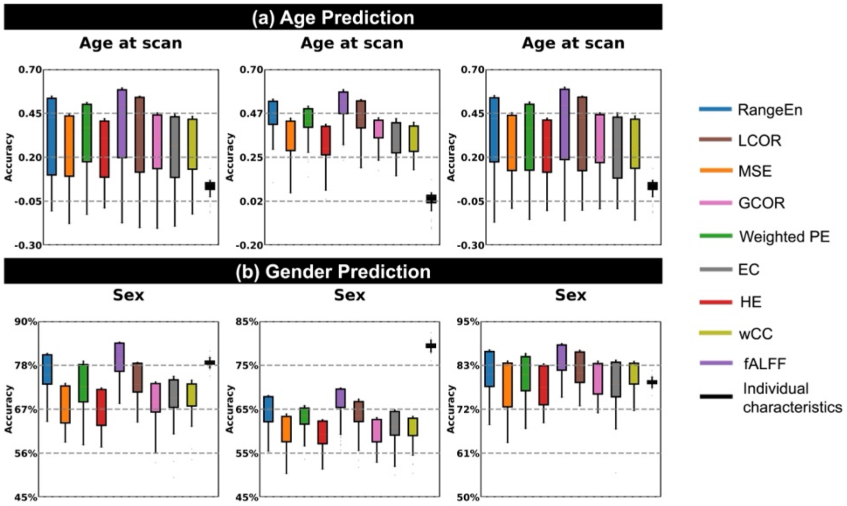

Figure S14: Prediction accuracy scores associated with nine rsfMRI features and age and gender as targets using scenarios 1–3 of this study using the Glasser360 brain atlas and linear SVM predictive modeling (see also figures 1-b.1–b.3 and Methods). For age prediction, we considered gender and TIV as confounds, while for gender prediction, we considered age and TIV as confounds. Age prediction accuracies are computed as the Pearson correlation between the actual values and predicted values through SVM modeling. Gender prediction accuracies are computed as the balanced accuracy through SVM binary classification. Each rsfMRI feature is illustrated in a distinct color and listed in the figure legend. In each figure panel, the box has a line at the median and spans the complete range of sample sizes (from 100 to 20,000 participants), extending from the lower to upper quartile values of the prediction accuracies. The whiskers extend outside the box to display the data's range. The population sizes from 100 to 2000 were increased with a 50-step increment and from 2000 to 20,000 with a 500-step increment. See figure S6 for the representation of prediction accuracies over the range of sample sizes. Abbreviations: rsfMRI = resting state functional magnetic resonance imaging, TIV = total intracranial volume, RangeEn = range entropy, MSE = multiscale entropy, wPE = weighted permutation entropy, HE = Hurst exponent, fALFF = fractional amplitude of low frequency fluctuations, LCOR = local correlation, GCOR = global correlation, EC = eigenvector centrality, wCC = weighted clustering coefficient, SVM = support vector machine.

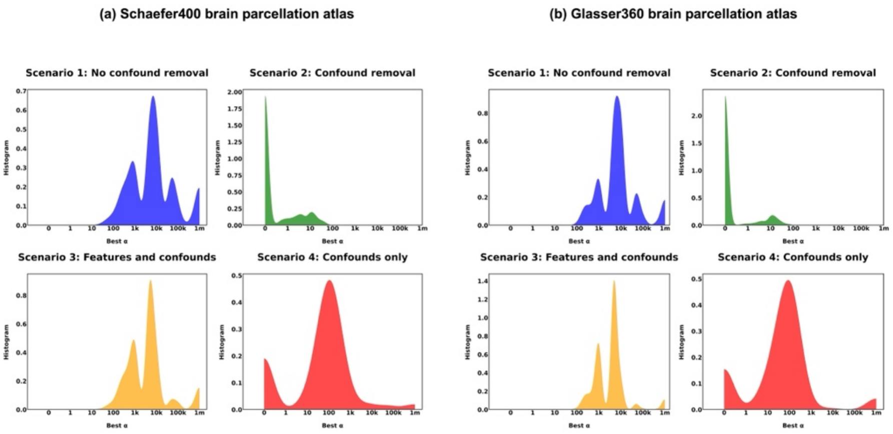

Figure S15: Optimal alpha parameter of the ridge models in four confound removal scenarios for rsfMRI feature vectors generated by (a) Schafer brain atlas with N_{ROI}=400, and (b) Glasser360 brain atlas with N_{ROI}=360. The distributions are associated with the rsfMRI feature vectors at no tSNR thresholding, i.e., utilizing complete number of ROIs in each brin atlas.

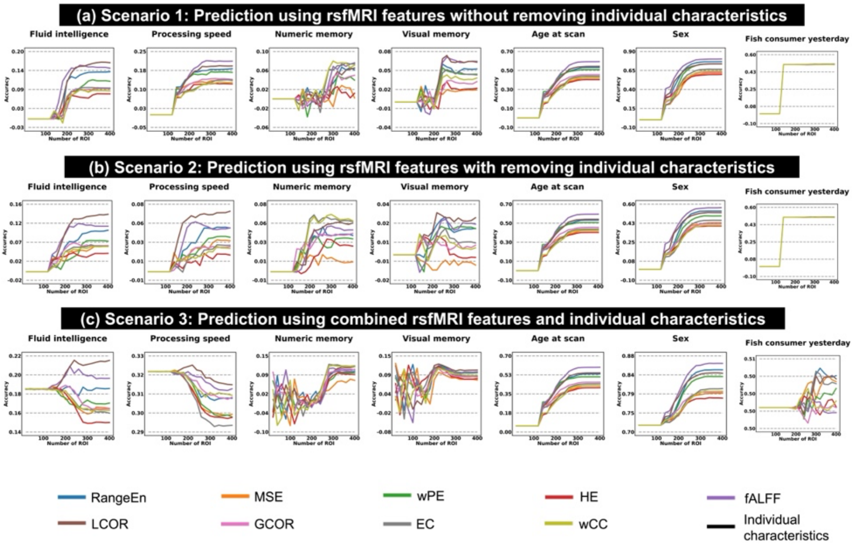

Figure S16: Prediction accuracy scores associated with nine rsfMRI features and five prediction targets using scenarios 1–3 of this study for the population size of $N_{subject}$ = 20,000 using the Schaefer400 brain atlas and linear SVM predictive modeling (see also figures 1-b.1–b.3 and Methods). Prediction accuracies of the fluid intelligence, processing speed, visual memory, and numeric memory scores are computed as the Spearman correlation between the actual values and predicted values through SVM modeling. The prediction accuracy of Fish consumer yesterday is computed as the balanced accuracy through SVM binary classification. Each rsfMRI feature is illustrated in a distinct color and listed in the figure legend. In each figure panel, the x-axis represents the number of suprathreshold ROIs after tSNR thresholding from 0% to 100%, and the y-axis shows the prediction accuracy. Abbreviations: rsfMRI = resting state functional magnetic resonance imaging, RangeEn = range entropy, MSE = multiscale entropy, wPE = weighted permutation entropy, HE = Hurst exponent, fALFF = fractional amplitude of low frequency fluctuations, LCOR = local correlation, GCOR = global correlation, EC = eigenvector centrality, wCC = weighted clustering coefficient, SVM = support vector machine.

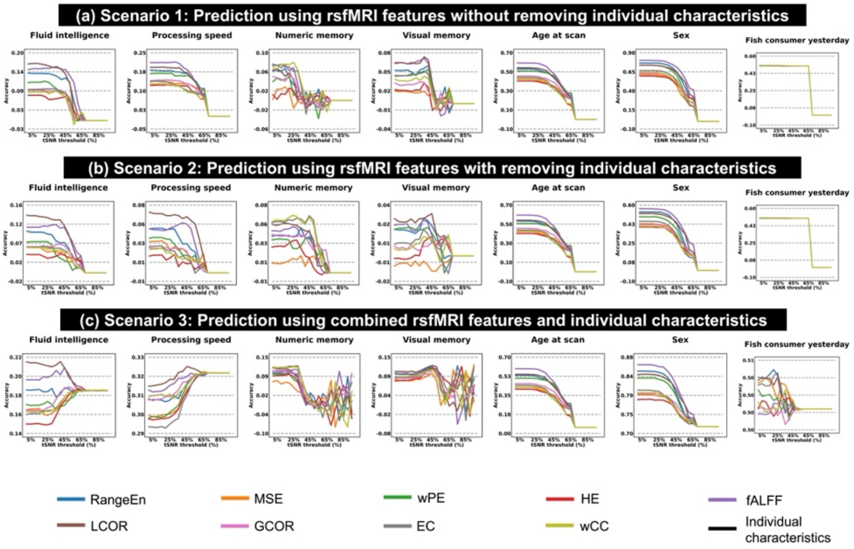

Figure S17: Prediction accuracy scores associated with nine rsfMRI features and five prediction targets using scenarios 1–3 of this study for the population size of $N_{subject}$ = 20,000 using the Schaefer400 brain atlas and linear SVM predictive modeling (see also figures 1-b.1–b.3 and Methods). Prediction accuracies of the fluid intelligence, processing speed, visual memory, and numeric memory scores are computed as the Spearman correlation between the actual values and predicted values through SVM modeling. The prediction accuracy of Fish consumer yesterday is computed as the balanced accuracy through SVM binary classification. Each rsfMRI feature is illustrated in a distinct color and listed in the figure legend. In each figure panel, the x-axis represents the tSNR thresholding levels, applied on the rsfMRI feature vectors, from 0% to 100%, and the y-axis shows the prediction accuracy. Abbreviations: rsfMRI = resting state functional magnetic resonance imaging, RangeEn = range entropy, MSE = multiscale entropy, wPE = weighted permutation entropy, HE = Hurst exponent, fALFF = fractional amplitude of low frequency fluctuations, LCOR = local correlation, GCOR = global correlation, EC = eigenvector centrality, wCC = weighted clustering coefficient, SVM = support vector machine.

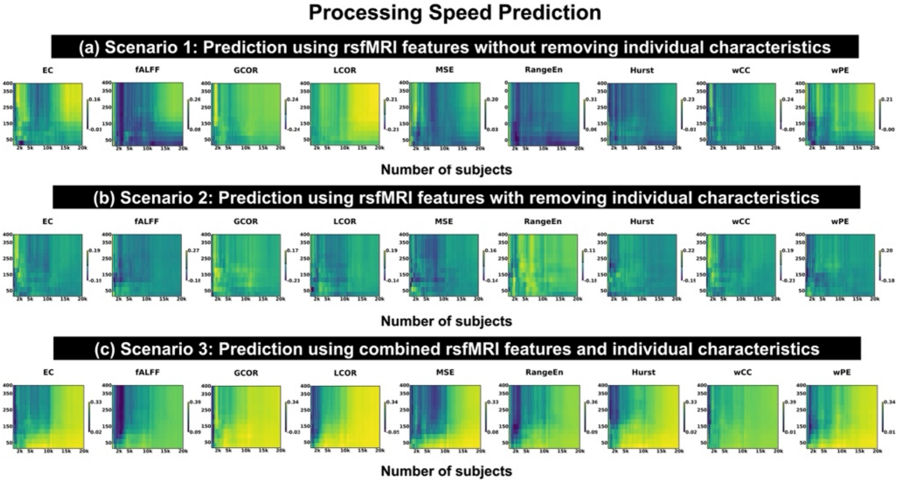

Figure S18: Spearman correlations associated with the Schaefer400 brain atlas and ridge regression modeling of the processing speed score using nine rsfMRI features and after tSNR thresholding from 0% (no threshold) to 100%. In each figure panel, the accuracy values are color-coded. Additionally, the x-axis represents the population size in the analysis, and the y-axis shows the number of suprathreshold ROIs after tSNR thresholding. The predictive modeling of each pair of features and targets is repeated for different sample sizes in the UK Biobank, ranging from $N_{subject}$ = 100 to $N_{subject}$ = 20,000. The population sizes from 100 to 2000 were increased with a 50-step increment, and from 2000 to 20,000 with a 500-step increment. Abbreviations: rsfMRI = resting state functional magnetic resonance imaging, tSNR = temporal signal to noise ratio, ROI = region of interest, RangeEn = range entropy, MSE = multiscale entropy, wPE = weighted permutation entropy, HE = Hurst exponent, fALFF = fractional amplitude of low frequency fluctuations, LCOR = local correlation, GCOR = global correlation, EC = eigenvector centrality, wCC = weighted clustering coefficient.

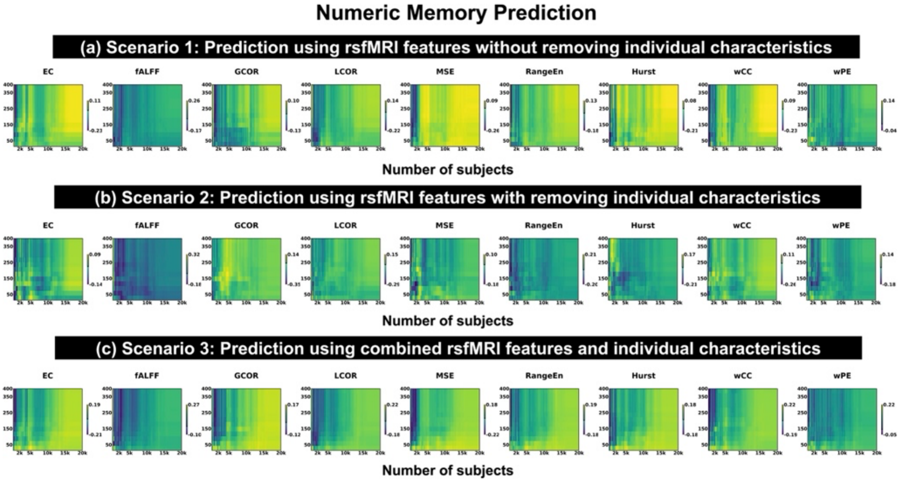

Figure S19: Spearman correlations associated with the Schaefer400 brain atlas and ridge regression modeling of the numeric memory score using nine rsfMRI features and after tSNR thresholding from 0% (no threshold) to 65%. In each figure panel, the accuracy values are color-coded. Additionally, the x-axis represents the population size in the analysis, and the y-axis shows the number of suprathreshold ROIs after tSNR thresholding. The predictive modeling of each pair of features and targets is repeated for different sample sizes in the UK Biobank, ranging from $N_{subject}$ = 100 to $N_{subject}$ = 20,000. The population sizes from 100 to 2000 were increased with a 50-step increment, and from 2000 to 20,000 with a 500-step increment. Abbreviations: rsfMRI = resting state functional magnetic resonance imaging, tSNR = temporal signal to noise ratio, ROI = region of interest, RangeEn = range entropy, MSE = multiscale entropy, wPE = weighted permutation entropy, HE = Hurst exponent, fALFF = fractional amplitude of low frequency fluctuations, LCOR = local correlation, GCOR = global correlation, EC = eigenvector centrality, wCC = weighted clustering coefficient.

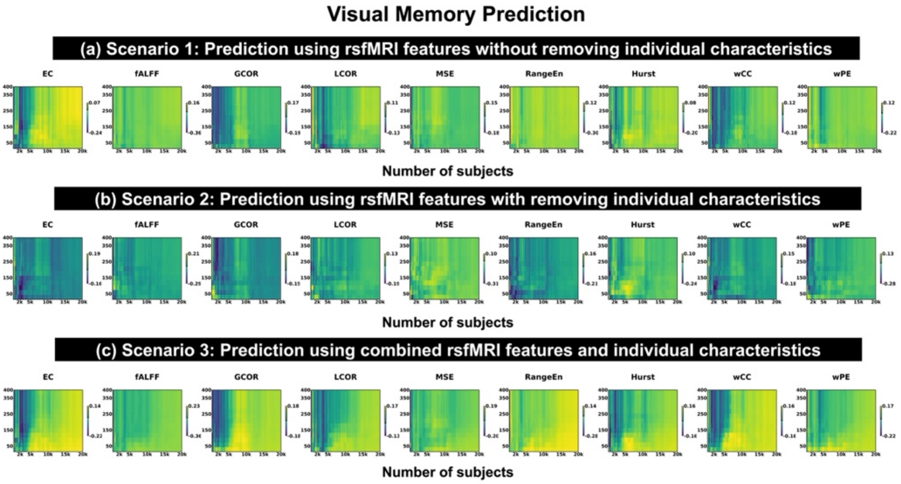

Figure S20: Spearman correlations associated with the Schaefer400 brain atlas and ridge regression modeling of the visual memory score using nine rsfMRI features and after tSNR thresholding from 0% (no threshold) to 65%. In each figure panel, the accuracy values are color-coded. Additionally, the x-axis represents the population size in the analysis, and the y-axis shows the number of suprathreshold ROIs after tSNR thresholding. The predictive modeling of each pair of features and targets is repeated for different sample sizes in the UK Biobank, ranging from $N_{subject}$ = 100 to $N_{subject}$ = 20,000. The population sizes from 100 to 2000 were increased with a 50-step increment, and from 2000 to 20,000 with a 500-step increment. Abbreviations: rsfMRI = resting state functional magnetic resonance imaging, tSNR = temporal signal to noise ratio, ROI = region of interest, RangeEn = range entropy, MSE = multiscale entropy, wPE = weighted permutation entropy, HE = Hurst exponent, fALFF = fractional amplitude of low frequency fluctuations, LCOR = local correlation, GCOR = global correlation, EC = eigenvector centrality, wCC = weighted clustering coefficient.

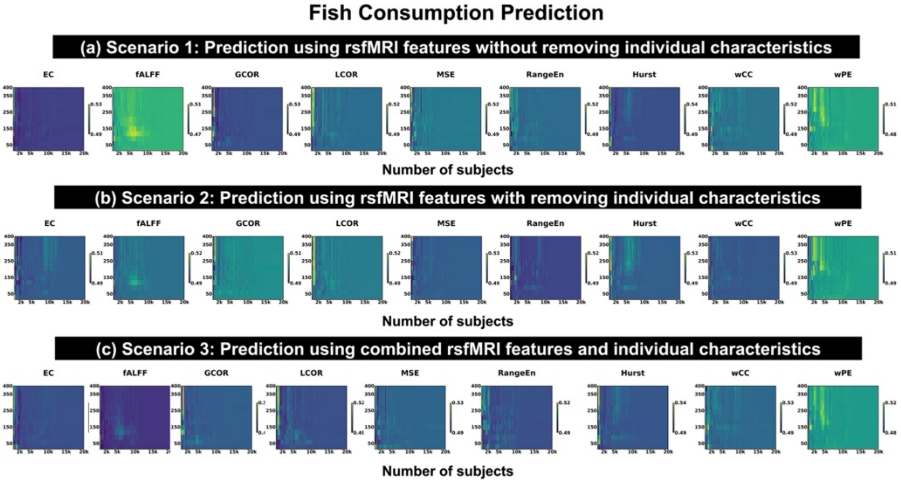

Figure S21: Spearman correlations associated with the Schaefer400 brain atlas and ridge regression modeling of the fish consumption yesterday using nine rsfMRI features and after tSNR thresholding from 0% (no threshold) to 65%. In each figure panel, the accuracy values are color-coded. Additionally, the x-axis represents the population size in the analysis, and the y-axis shows the number of suprathreshold ROIs after tSNR thresholding. The predictive modeling of each pair of features and targets is repeated for different sample sizes in the UK Biobank, ranging from $N_{subject}$ = 100 to $N_{subject}$ = 20,000. The population sizes from 100 to 2000 were increased with a 50-step increment, and from 2000 to 20,000 with a 500-step increment. Abbreviations: rsfMRI = resting state functional magnetic resonance imaging, tSNR = temporal signal to noise ratio, ROI = region of interest, RangeEn = range entropy, MSE = multiscale entropy, wPE = weighted permutation entropy, HE = Hurst exponent, fALFF = fractional amplitude of low frequency fluctuations, LCOR = local correlation, GCOR = global correlation, EC = eigenvector centrality, wCC = weighted clustering coefficient.

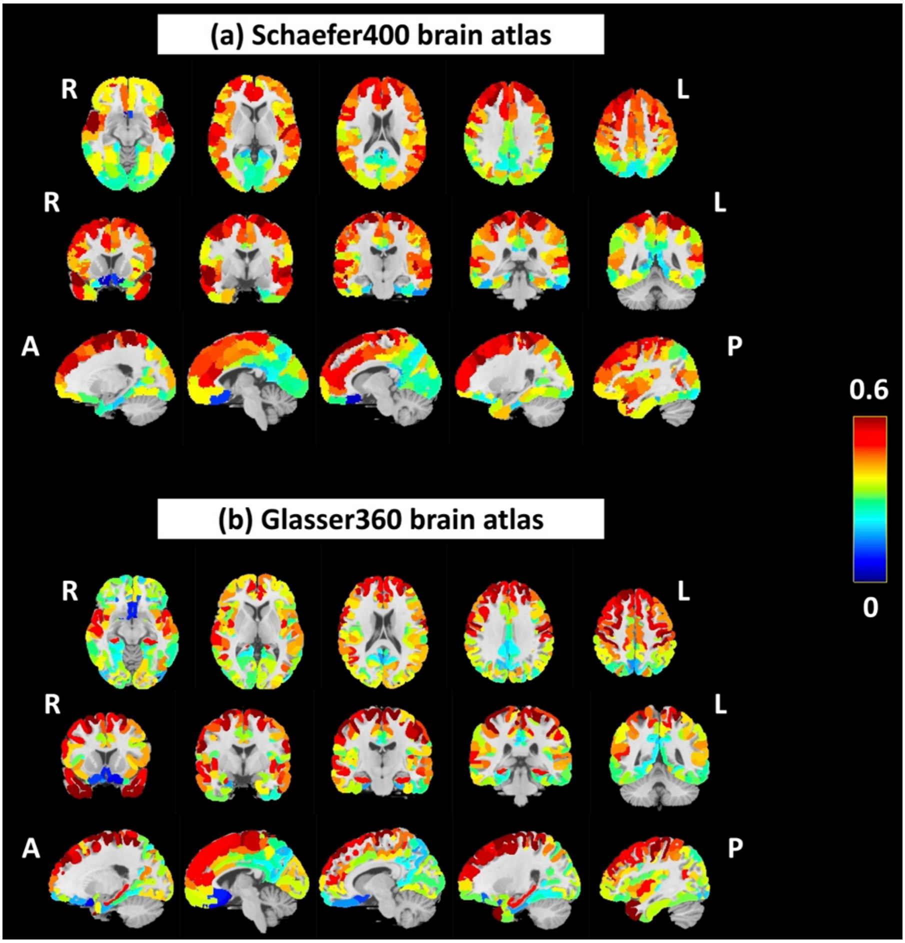

Figure S22: Group level mean tSNR brain maps obtained using the (a) Schaefer400 and (b) Glasser360 brain atlases. The maps have been averaged after min-max normalization of the subject-specific tSNR maps over the entire population of 20,000 UK Biobank subjects. The numbers of suprathreshold ROIs at tSNR threshold level spanning from 0% to 60% with 5% increment for the Schaefer and Glasser brain atlases are [400, 397, 397, 396, 387, 366, 333, 276, 201, 130, 68, 33, 13] and [360, 360, 356, 353, 338, 311, 270, 203, 142, 83, 57, 25, 10], respectively. For both brain atlases, tSNR levels above 60% led to no suprathreshold ROIs. Abbreviations: rsfMRI = resting state functional magnetic resonance imaging, tSNR = temporal signal to noise ratio, ROI = region of interest, L = left, R = right, A = anterior, P = posterior.
